# Extracellular electrons transferred from honey probiotic *Bacillus circulans* inhibits inflammatory acne vulgaris

**DOI:** 10.1038/s41598-022-23848-9

**Published:** 2022-11-10

**Authors:** Hsin-Jou Kao, Arun Balasubramaniam, Chun-Chuan Chen, Chun-Ming Huang

**Affiliations:** Department of Biomedical Sciences and Engineering, National Central University, No. 300, Zhongda Rd., Zhongli District, Taoyuan, 32001 Taiwan R.O.C.

**Keywords:** Microbiology, Microbiology techniques

## Abstract

*Bacillus circulans* (*B. circulans*) is widely used as an electrogenic bacterium in microbial fuel cell (MFC) technology. This study evaluated whether *B. circulans* can ferment glucose to generate electricity and mitigate the effects of human skin pathogens. The electricity production of *B. circulans* was examined by measuring the voltage difference and verified using a ferrozine assay in vitro. To investigate the fermentation effects of *B. circulans* on inhibition of human skin pathogens, *Cutibacterium acnes* (*C. acnes*) was injected intradermally into mice ears to induce an inflammatory response. The results revealed that the glucose–*B. circulans* co-culture enhanced electricity production and significantly supressed *C. acnes* growth. The addition of roseoflavin to inhibit flavin production considerably reduced the electrical energy generated by *B. circulans* through metabolism and, in vivo test, recovered *C. acnes* count and macrophage inflammatory protein 2 (MIP-2) levels. This suggests that *B. circulans* can generate electrons that affect the growth of *C. acnes* through flavin-mediated electron transfer and alleviate the resultant inflammatory response. Our findings demonstrate that probiotics separated from natural substances and antimicrobial methods of generating electrical energy through carbon source fermentation can help in the treatment of bacterial infections.

## Introduction

Honey is a naturally sweet substance, mainly consisting of glucose and fructose. Many studies have reported honey to have antibacterial activity and to be effective in wound management^1^. However, some beneficial microorganisms have been found in honey in the form of spores to resist high concentrations of acids and sugar^1^. Nonpathogenic bacterial strains in honey can also grow when honey is diluted with water^2^. These nonpathogenic microorganisms include yeast (1%) and gram-positive bacteria (27%) such as *Bacillus* was one of the predominant genera^3,4^. Previous study reported that *B. circulans* are present in the digestive tracts of honey bees and can inhibit the growth of *Ascophaera apis,* the causative agent of chalkbrood disease in honeybee larvae, possibly through bacteriocins and other antimicrobial molecules^4^. Additionally, microorganisms such as *Bacillus subtilis*^5^, and *Clostridium butyricum*^6^ can produce bioelectricity through extracellular electron transfer (EET). *B. circulans* is an electrogenic bacterium^7^ with potential for application in MFC technology. In MFCs, *Bacillus cereus* strain DIF1 and *Rhodococcus ruber* strain DIF2 actively secrete riboflavin and flavin mononucleotide (FMN), which contribute as electron mediators in EET, mediate electron transfer to extracellular acceptors, and enhance electric current production^8^. Furthermore, through the addition of exogenetic flavins, *Bacillus megaterium* (*B. megaterium*) strain LLD-1 can increase the production of electricity by the fermentation of different carbon sources^9^. In short, *B. circulans* can be isolated from honey and generate electricity.

During metabolism and EET, NADH is oxidised to NAD^+^ through NADH dehydrogenase and delivers electrons to the extracellular space to reduce extracellular electron acceptors^10^. EET includes direct and indirect modes, such as conductive protein filaments (microbial nanowires^11^), electron-shuttling mediators (flavin^12^ or methyl viologen^13^), and the extracellular polymeric substances of biofilms (redox proteins^14^). Recently, flavins that mediate EET have received more attention. Flavins are common cofactors that are highly effective as redox enzymes in natural biological systems. In catalytic reactions, flavins oxidise electron donors, such as hydrogen and bacterial fermentation products, and release electrons. These electrons are then used to reduce extracellular electron acceptors such as Fe(III) or Mn(IV)^15–17^.

Acne vulgaris is a skin disease in which the skin commensal *C. acnes* overcolonises the pilosebaceous unit and secretes lipase. The lipase breaks down triglycerides to release free fatty acids and stimulates the cells to produce proinflammatory cytokines, including interleukin (IL)-8, IL-12^18^, IL-1β^19^, and MIP-2^20^, resulting in severe inflammation^21^. Acne is most commonly treated through antibiotic application, which inhibits *C. acnes* overgrowth and lipase activity; however, this therapy has several side effects, such as promoting the emergence of antibiotic‐resistant *C. acnes* strains and nonspecific killing of other skin commensal bacteria^22^. Alternatively, we reported that short-chain fatty acids (SCFAs), the fermentation metabolite from *Staphylococcus epidermidis* (*S. epidermidis*) could inhibit the growth of *C. acnes*. The injection of *S. epidermidis* with sucrose in an animal model led to decreases in MIP-2 levels and *C. acnes*-induced inflammation^23^. In the present study, we further evaluated whether the honey probiotic *B. circulans* can ferment glucose to generate electricity, thereby reducing *C. acnes* lipase-induced MIP-2 levels, in addition to supressing *C. acnes* growth, through flavin-based EET.

## Methods

### Ethics statement

This study was carried out in strict with an approved Institutional Animal Care and Use Committee (IACUC) protocol at National Central University (NCU), Taiwan (NCU-106-016) and in compliance with the Arrive guidelines (https://arriveguidelines.org/). Institute Cancer Research (ICR) mice aged 8–9 weeks females (National Laboratory Animal Centre, Taipei, Taiwan) were sacrificed under CO_2_ anesthesia in a sealed chamber. All methods were performed in accordance with relevant guidelines and regulations.

### Bacterial culture

The wildflower honey (Neu Wang Feng Co., Ltd., Taoyuan, Taiwan) was diluted 1:10 with PBS and incubated at 37 °C on TSB agar plate. After 3 days, bacteria were collected and analyzed by 16S rRNA gene sequencing (Tri-I Biotech Inc., New Taipei, Taiwan). Three bacteria were identified as *B. circulans*, *Lysinibacillus fusiformis* (*L. fusiformis*), and *Bacillus asahii* (*B. asahii*). *C. acnes* (ATCC 6919) and *B. circulans* were cultured on Reinforced Clostridium Medium (RCM, BD, Sparks, MD, USA) under anaerobic conditions using a Gas-Pak (BD) and Tryptone Soy Broth (TSB, BD) medium. Bacteria were cultured at 37 °C until the logarithmic growth phase. Bacterial pellets were harvested by centrifugation at 5000 × g for 10 min, washed in phosphate-buffered saline (PBS), and then suspended in PBS for further experiments.

### Bacteria fermentation

*B. circulans* (10^7^ colony-forming unit (CFU)/mL) was incubated in 5 mL TSB in the in the presence or absence of 2% glucose at 37 °C. Glucose alone in TSB was included as control. The 0.002% (w/v) phenol red (Sigma, Burlington, MA, USA) in TSB served as fermentation indicator. A colour changes from red–orange to yellow indicated the occurrence of bacterial fermentation, which was detected by optical density at 560 nm (OD560).

### Electricity detection by MFC system

MFC compartments was established for detection of bacterial electricity. The carbon cloth (9 × 9 cm) (Homy Tech, Taoyuan, Taiwan) as cathode, carbon filth (2.5 × 5 cm) (Homy Tech, Taoyuan, Taiwan) as anode and proton exchange membrane reated by Nafion membrane N117 (5 × 5 cm) (Homy Tech, Taoyuan, Taiwan). Anode and cathode were linked by copper wires, which in turn were connected to 1000 Ω external resistance. Bacteria (10^7^ CFU) with and without 2% glucose or 0.1 μM roseoflavin (flavin inhibitor) in TSB media was loaded on the surface of anode. The cell voltage was recorded every 30 s by a digital multimeter (DM-9962SD, Lutron, Australia) for 20 min.

### Ferrozine assay

Ferrozine assay was performed by suspending *B. circulans* (10^7^ CFU) in TSB medium with and without 2% glucose and 0.1 μM roseoflavin total 50 μl, an equal volume of ferrozine (8 mM) (Sigma) and 100 μl ferric ammonium citrate (100 mM) (Sigma) were added into each well. The mixture was incubated at 37 °C for 1 h in 96-well. The colour change of media was detected from OD at 562 nm.

### Electrons from *B. circulans* fermentation with glucose inhibits *C. acnes* growth in vitro and in the presence of roseoflavin in vivo

In vitro, Co-Culture *C. acnes* (10^7^ CFU) and *B. circulans* (10^7^ CFU) in TSB with and without 2% glucose under anaerobic conditions for 3 days at 37 °C. *B. circulans* and glucose alone mix with *C. acnes* was included as a control. After 3 days, dilute with PBS for *C. acnes* bacterial counts. In vivo, the ears of ICR mice were injected intradermally with *C. acnes* (10^7^ CFU) mix with *B. circulans* and 2% glucose in the presence or absence of 0.1 μM roseoflavin. *B. circulans* and glucose mix with *C. acnes* was included as a control. After 3 days, cut the mice ears and homogenized for *C. acnes* bacterial counts.

### Bacteria counting

The *C. acnes* loads in in vitro and in vivo sample were enumerated by plating serial dilutions (1:10–1:10^5^) with PBS of selective agar plates containing RCM media and 10 μg/mL of furazolidone (Sigma). The plates were incubated for 5 days at 37 °C in an anaerobic chamber using Gas-Pak.

### Cloning of Lipase

Transformation of a plasmid encoding lipase (accession number: YP_056770.1) into *Escherichia coli* (*E. coli*) BL21 competent cells (Invitrogen, Carlsbad, CA, USA). The *E. coli* BL21 transformed with a plasmid encoding GFP was used as a control by following the same procedure. A transformant of *E. coli* BL21 was inoculated with Luria–Bertani (LB) (Biokar Diagnostics, Beauvais, France) medium containing ampicillin (Sigma) at 37 °C until the OD600 reached 0.6–0.8. 1 mM Isopropyl-B-D-thiogalactoside (IPTG) (Sigma, Burlington, MA, USA) was added into culture for 4 h at 30 °C to induce protein expression. Proteins were purified by ProBond™ Purification System (Invitrogen, Carlsbad, CA, USA).

### *C. acnes* lipase-induced the proinflammatory MIP-2 cytokine and the treatment of electrons from *B. circulans* fermented by glucose in vivo

The ears of ICR mice were injected intradermally with 5/10 μl lipase or GFP (as a control) to induces the inflammation. After 24 h, cut the mice ears and the level of MIP-2 cytokine was measured by ELISA. In electrons treatment experiment, the ears of ICR mice were injected intradermally with 5/10 μl lipase to induces the inflammation. After 24 h, *B. circulans*, 2% glucose with 0.1 μM roseoflavin was injected. *B. circulans* and glucose without roseoflavin was included as a control. After 24 h, cut the mice ears and homogenized for MIP-2 quantified.

### ELISA

The proinflammatory MIP-2 cytokines in the supernatants of ear homogenates was quantified by an ELISA kit, as directed by the manufacturer (R&D System. Inc., Minneapolis, MN, USA).

### Statistical analyses

Data analysis was performed by unpaired t-test using Prism software (https://www.graphpad.com/; Version 5.01, GraphPad Software, La Jolla, CA, USA). The levels of statistical significance were indicated as the following: *p < 0.05, **p < 0.01, ***p < 0.001 and ns = non-significant. The mean ± standard.

deviation (SD) for at least three independent experiments was calculated. Animal experiments were performed with at least three animals per each treatment group.

## Results

### Bacteria sequencing from honey

By using 16S rRNA gene analysis, we isolated three bacteria (Supplementary Fig. S1A), *B. circulans*, *L. fusiformis*, and *B. asahii* (Supplementary Table S1) from TSB agar plate with 10% honey. Particularly, one 16S rRNA gene sequence of the isolated three bacteria shares 99% similarity to that of *B. circulans* strain FDAARGOS_783 (GenBank accession no NZ_CP053989.1).

### Electricity production and electron transfer by *B. circulans* produced through glucose fermentation

**Figure 1:**
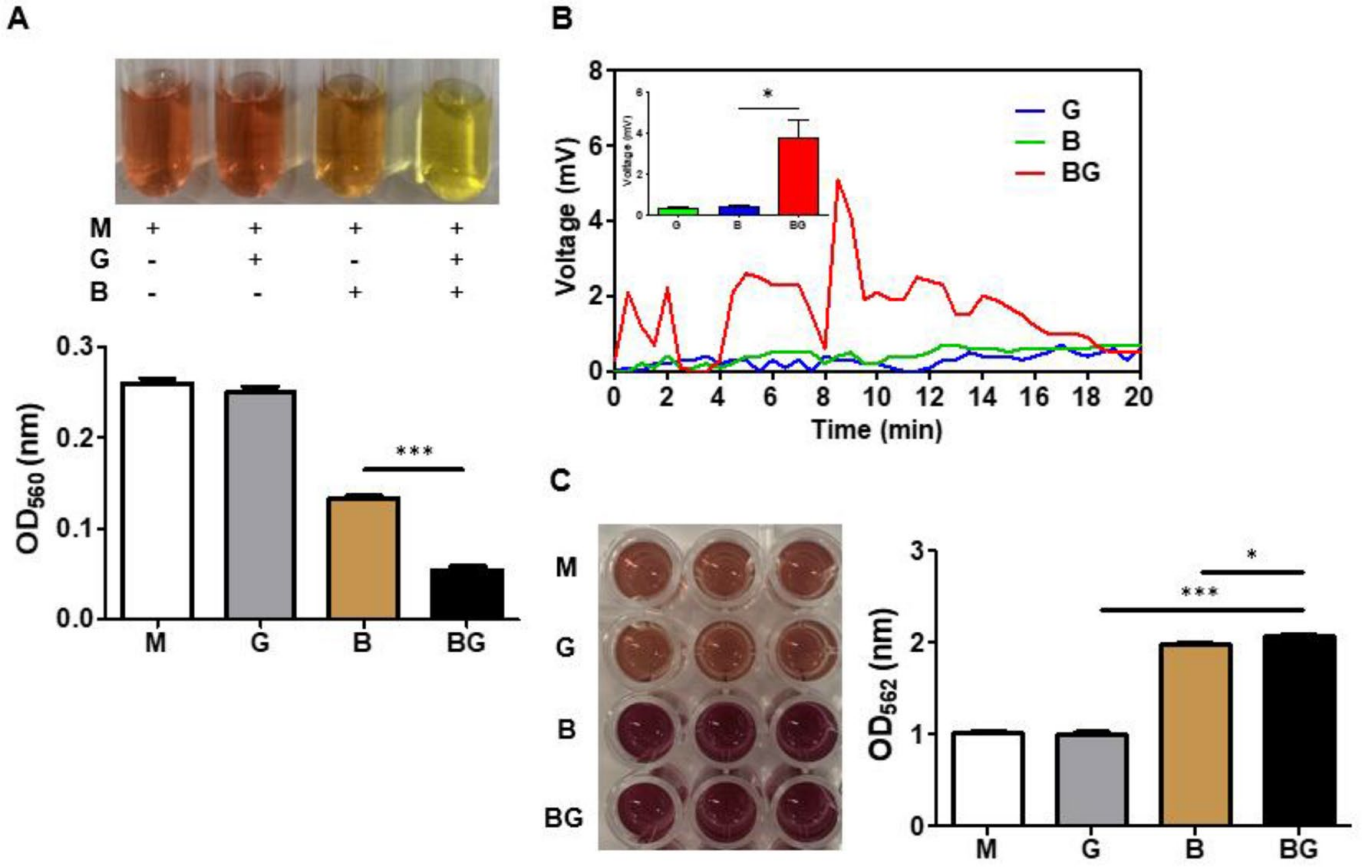
Electricity production and electron transfer by *B. circulans* produced through glucose fermentation. (**A**) The colour change of phenol red when *B. circulans* (B) was incubated with/without glucose (G) in TSB medium (M). The OD560 in the media with glucose and *B. circulans* (BG) was significantly lower than the other groups. (**B**) Voltage changes detection (mV) for 20 min in medium alone or media containing glucose, *B. circulans*, or *B. circulans* plus glucose. (**C**) The concentration of ferrozine-chelatable iron (mM) in medium or media containing glucose, *B. circulans*, or *B. circulans* plus glucose. Data are expressed as the mean ± SD of three separate experiments. *p < 0.05 and ***p < 0.001.

To investigate whether *B. circulans* can ferment glucose, it was incubated with 2% glucose in TSB media with phenol red for 1 day. With *B. circulans* alone, phenol red colour changed to orange because of bacterial replication during incubation. However, when *B. circulans* was incubated along with glucose, phenol red colour changed to yellow with a decrease in pH value, indicating the use of glucose for fermentation (Fig. 1A; upper panel). Furthermore, the quantification of fermentation by measuring the optical density of phenol red at OD560 nm indicated a significant decrease of OD560 values in TSB media containing bacteria plus glucose medium compared with bacteria or glucose alone (Fig. 1A; lower panel).

Next, electricity production in the *B. circulans* with glucose was identified by adding the fermented media to the anode of the MFC system. *B. circulans* and glucose alone were used as controls and exhibited a low voltage change at 20 min (Fig. 1B; blue and green lines). By contrast, the voltage significantly increased to approximately 4 mV in the *B. circulans* with 2% glucose group (Fig. 1B; red line). We further verified that *B. circulans* can produce electrons through glucose fermentation by using the ferrozine assay to identify ferric iron reductase activity. In Fig. 1C, it can be seen that the concentration of ferrozine-chelatable iron (dark brown) in the reaction solution containing a fermentation medium of *B. circulans* plus glucose was distinctly higher than the medium, glucose, or bacteria alone (Fig. 1C; left panel). Statistical tests further confirmed that the ferric iron reductase activity of *B. circulans* was significantly increased when with glucose than without glucose (Fig. 1C; right panel). When compared with glucose alone*, B. circulans* and glucose resulted in significant increase in OD value.

### Roseoflavin affects electricity production by *B. circulans* fermenting glucose

**Figure 2:**
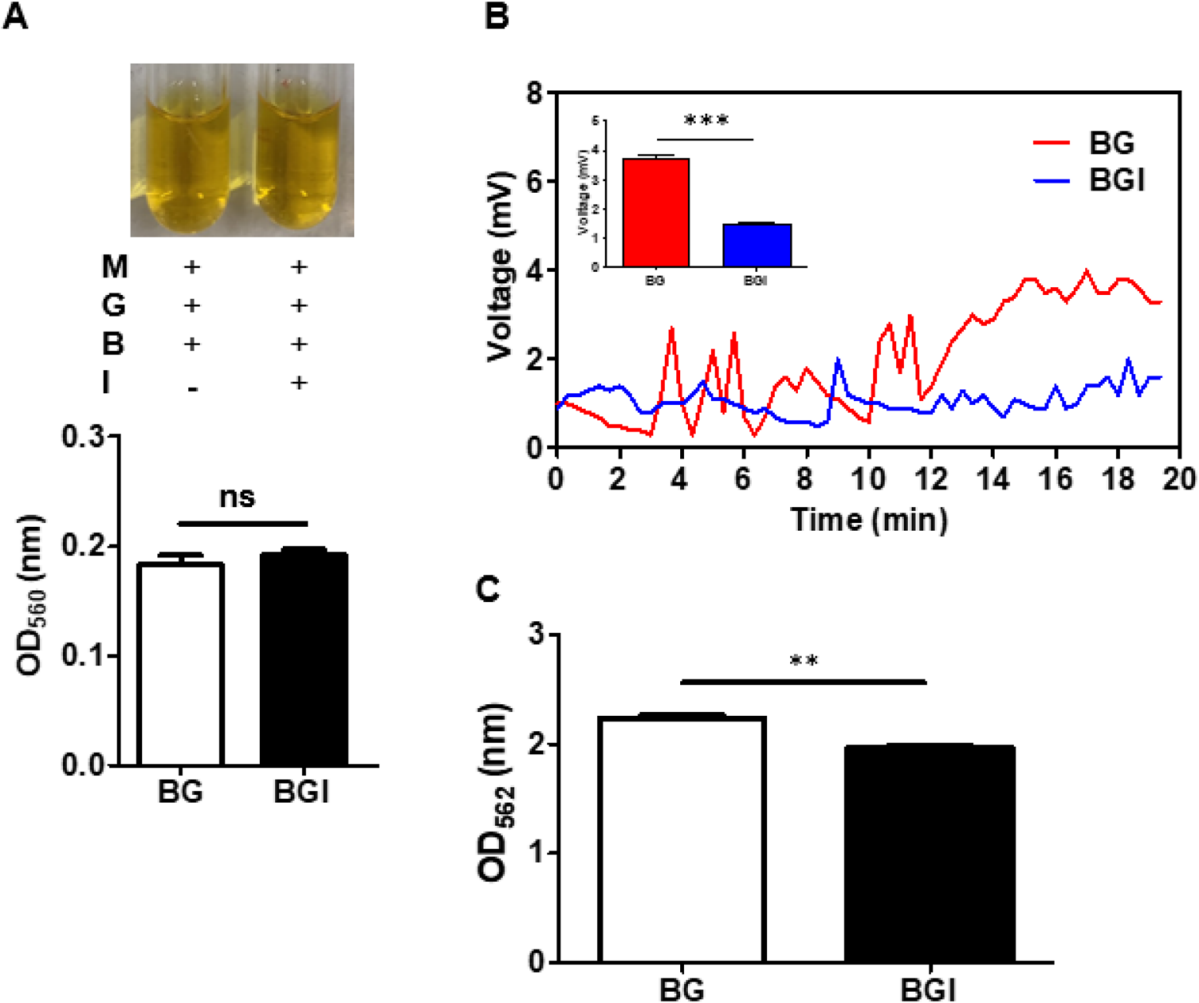
Roseoflavin affects electricity production in the fermentation of *B. circulans* with glucose. (**A**) *B. circulan*s with glucose in the presence and absence of roseoflavin (I) in TSB medium with phenol red. The colour change of phenol red in media from red to yellow indicated fermentation still occurred. Furthermore, fermentation was quantified by measuring the optical density of phenol red at OD560. (**B**) Voltage change detection (mV) for 20 min in the culture of *B. circulans* with glucose in the absence or presence of roseoflavin. (**C**) The concentration of ferrozine-chelatable iron (mM) in the media in the absence or presence of roseoflavin. Data are expressed as the mean ± SD of three separate experiments. **p < 0.01 and ns = non-significant.

Gram-positive bacteria used flavin-based EET to deliver electrons^2^. Roseoflavin represses FMN riboswitch and subsequently mediates riboflavin and FMN gene expression^24^. In Fig. 2A, 0.1 µM roseoflavin was added to the culture of bacteria and glucose, and phenol red colour changed to yellow with a decrease in pH, similar to that in the fermentation experiment illustrated in Fig. 1A, confirming that roseoflavin does not affect the fermentation of *B. circulans*. Next, to identify electricity production in the *B. circulans*-glucose-roseoflavin culture, the fermented medium was added to the anode of the MFC system. The voltage change induced by *B. circulans* in the presence of glucose was completely attenuated by the addition of roseoflavin (Fig. 2B). In Fig. 2C, the concentration of ferrozine-chelatable iron in the reaction solution containing the fermentation medium of *B. circulans*-glucose-roseoflavin was distinctly decreased compared with medium, glucose, and bacteria. In summary, the findings of similar acidities but significantly decreased voltage production when adding roseoflavin indicated that the voltage change was not due to the pH change during fermentation and the number of *B. circulans* because 0.1 μM roseoflavin did not influence bacterial growth (Supplementary Fig. S2 for details).

### *B. circulans* fermentation with glucose affect *C. acnes* growth in vitro and in the presence of roseoflavin in vivo

**Figure 3:**
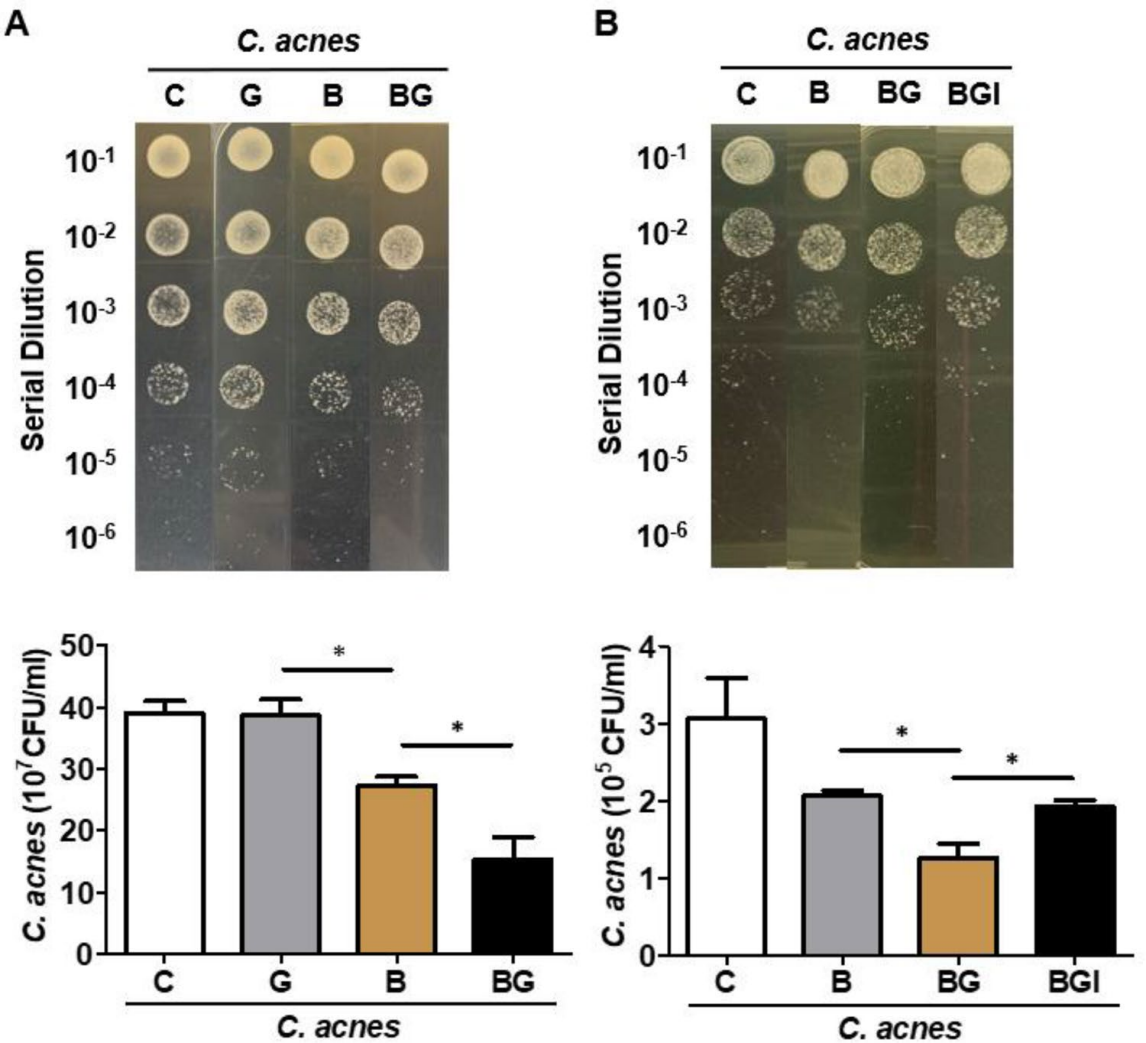
*B. circulans* fermentation with glucose affect *C. acnes* growth in vitro and in the presence of roseoflavin in vivo. (**A**) In vitro, the *C. acnes* (CFU) was assessed by enumerating a plating serial dilution (1:10–1:10^5^) on selective agar containing RCM agar plus furazolidone. (**B**) The CFU of *C. acnes* was assessed in *B. circulans* with glucose in the presence of roseoflavin (BGI) in vivo. *C. acnes* mix with PBS (C), glucose (G), *B. circulans* (B), and *B. circulans* plus glucose (BG) was included. Data are expressed as the mean ± SD of three separate experiments. *p < 0.05.

Having established the electricity produced in *B. circulans-*glucose cultures, we cocultured *C. acnes* and *B. circulans *in vitro to test the impact of *B. circulans* fermentation on the growth of *C. acnes.* The number of *C. acnes* was significantly decreased when mixed with *B. circulans* and glucose (Fig. 3A). Furthermore, the *B. circulans*-*C. acnes*-glucose mixture was injected intradermally with or without roseoflavin in ICR mice ears to examine whether glucose fermentation of *B. circulans* with roseoflavin changed the bacterial growth by electron. The result shows that the *C. acnes* count was significantly decreased in the absence of roseoflavin but increased in the presence of roseoflavin (Fig. 3B).

### *C. acnes* lipase induced proinflammatory MIP-2 cytokine and the treatment of electrons from *B. circulans* fermented by glucose in vivo

**Figure 4:**
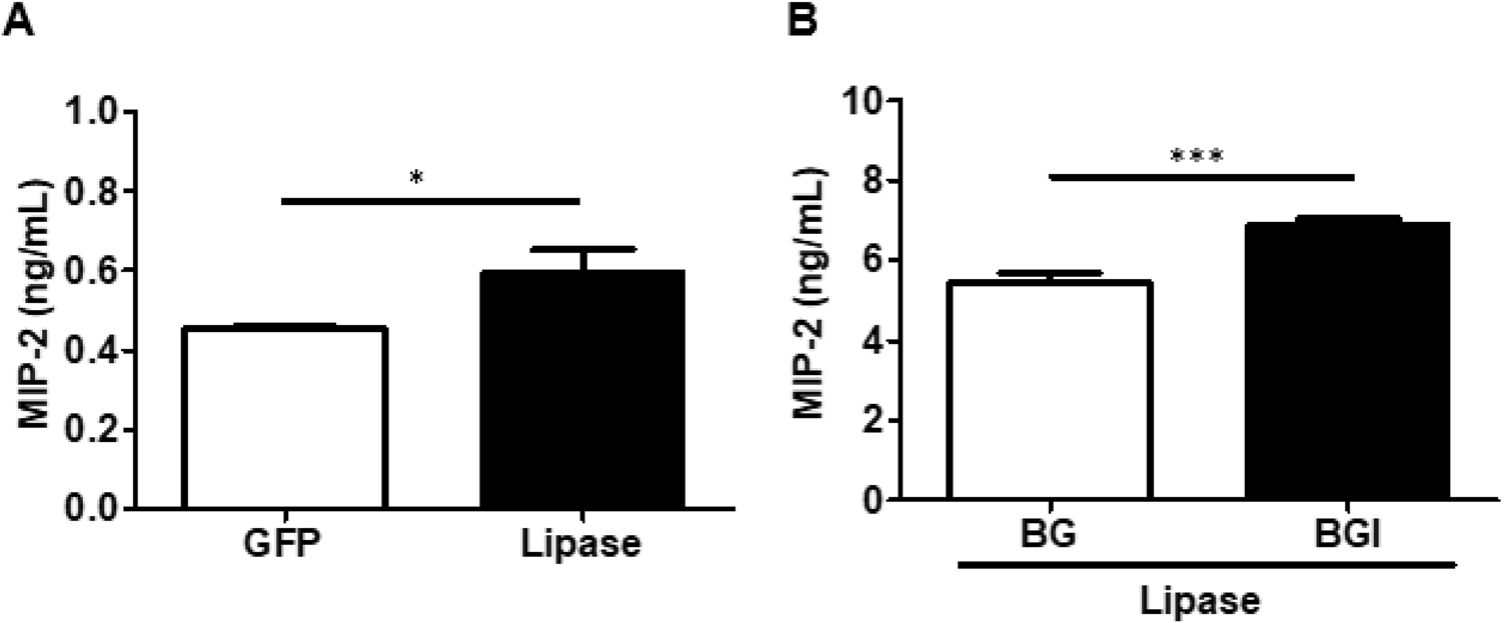
MIP-2 expression through electron treatment in mice ears after lipase injection. (**A**) MIP-2 expression (ng/mL) after lipase induction. (**B**) MIP-2 expression (ng/mL) in mouse ears of *B. circulans* with glucose in the absence (BG) or presence (BGI) of roseoflavin. Data are expressed as the mean ± SD from three separate experiments. *p < 0.05 and ***p < 0.001.

After 24 h induction of lipase injection (with GFP as the control), MIP-2 expression was elevated in the presence of lipase (Fig. 4A). In the electron treatment experiment, the use of roseoflavin dramatically decreased electricity production, attenuated the anti-inflammatory defence of *B. circulans* fermentation by glucose, and significantly increased the concentrations of lipase-induced MIP-2 (Fig. 4B).

## Discussion

Through probiotics’ fermentation of proper carbon sources, pathogen-caused skin diseases can be reduced. For instance, SCFAs, when used as fermentation metabolites, can inhibit bacterial growth to achieve treatment effects^25^. In addition, it has been shown that *B. circulans* can convert biomass into electrical energy^10^. Its halophilic strain BBL03 can ferment 1% chitin and use degraded metabolites as electron donors to generate electricity in seawater; therefore, it can serve as electricity-producing bacteria in MFCs^26^. In this study, after adding 2% glucose to the medium containing *B. circulans*, the electricity increased significantly when measured using changes in voltages, indicating that *B. circulans* can generate a substantial number of electrons through glucose fermentation. Notably, the low electricity was also detected in the medium containing *B. circulans* without the addition of glucose. The reason for this slight change of electricity production may be due to the presence of a small amount of glucose in TSB (Fig. 1B).

In gram-positive bacteria, EET is involved in iron redox. Fe^3+^-reducing microorganisms belonging to the Geobacteraceae family can ferment sugars and other organic compounds to produce simple organic acids (such as acetate) that serve as electron donors to the electrodes^27^. Similarly, by adding *B. circulans* and glucose to the solution containing ferric (Fe^3+^) ammonium citrate, we observed that the concentration of ferrozine-chelated Fe^2+^ was higher than that in the groups without glucose (Fig. 1C). The addition of glucose allowed more electrons to be generated, leading to increased ferric reduction, which rendered the culture medium-dark brown.

The flavin-based EET mechanism has been confirmed in various gram-positive bacteria. Flavin in the suspension culture of the *B. megaterium* LLD-1 strain acted as an electron shuttle, enhancing electron transfer from LLD-1 to the electrode^8^. Roseoflavin is a natural antibacterial compound. When combined with FMN riboswitch, it can inhibit Rli96 transcription, control the expression of downstream genes, and regulate the in vivo synthesis of flavin, thus impairing the bacterial metabolism and achieving bacteriostasis^28^. Roseoflavin can also be converted into roseoflavin mononucleotide and roseoflavin adenine dinucleotide, both of which cause defects in cellular physiological functions^29^. Therefore, FMN riboswitch may serve as a novel target for inhibiting pathogens. We used phenol red to monitor the degree of acid production by bacterial fermentation, and the addition of roseoflavin as flavin inhibitor to the culture medium did not alter the production of organic acids (Fig. 2A). This suggests that the reduction of electrons was not caused by organic acids but by the roseoflavin-induced inhibition of flavin generation. The ferrozine assay indicated that the decrease of iron concentration represented a decrease in electrons produced by fermentation (Fig. 2C).

We previously reported that *S. epidermidis* can ferment glycerol and PEG-8 laurate to generate potential electron donors for electricity generation to combat ultraviolet damage^30^ or suppress acne vulgaris^31^. In this study, we further demonstrated that adding glucose to the culture medium of *B. circulans *in vitro can inhibit the growth of *C. acnes*; specifically, this suppression was significantly reversed when roseoflavin was added to the mix. Taken together, *B. circulan* affected the growth of *C. acnes* through the electrons generated by glucose fermentation and flavin-mediated EET. These results also reveal an efficient EET mechanism to target pathogenic microorganisms by using electrons. Importantly, the intradermal application of *B. circulans* plus glucose to mice ears can suppress the *C. acnes* count while the use of nonselective anti-inflammatory drugs for treatment may lead to epidermal dysbiosis and the spread of resistant strains.

In acne pathogenesis, the increased activity of the virulence factor lipase caused by *C. acnes* overcolonisation led to an inflammatory response that resulted in the release of proinflammatory cytokines and TNF-α, which modulated host immune response^2,20^. In line with previous reports, lipase-induced immune responses were confirmed by an elevated MIP-2 content measured using ELISA in this study. Adding glucose to *B. circulans* can inhibit lipase-induced MIP-2 expression, but this inhibition was reversed when roseoflavin was also added. Therefore, *B. circulans* can generate electrons through glucose fermentation to affect the growth of *C. acnes* through flavin-mediated electron transfer, thereby reducing the resultant inflammatory response. In short, weak currents inhibit bacterial growth. The underlying mechanisms for the current-related lysis may be because of electron-induced electrolysis, the generation of free radicals, pH, and changes in biofilm structure^32^. It was reported that exposure of gram-positive bacteria to pulsed electric fields can induce permeabilization of the plasma membrane, destabilising the cell wall and causing osmotic shock^33^. In other words, electric current generated with conductivity electrodes can directly inhibit bacterial growth, but the transition of platinum complexes and metal ions generated during electrolysis can harm human cells^34^. By contrast, the weak current produced by *B. circulans* through glucose fermentation, as demonstrated in this study, can efficiently and safely supress pathogenic bacterial growth.

Overall, this study revealed the molecular mechanism by which the probiotic *B. circulans* in honey can generate electrical energy by using glucose as a prebiotic. *B. circulans* reduced the inflammatory response by disrupting *C. acnes* growth through FMN riboswitch and flavin-mediated electron transfer. Therefore, generating electrical energy from biomass through the metabolic activities of microorganisms may be a potential antimicrobial therapy. These results are beneficial for the future clinical treatment of acne-prone skin disorders and to development of skincare products.

## Supplementary Information

Supplementary Information.

## Data Availability

The datasets generated and analyzed during the current study available from the corresponding author on reasonable request.
