# Supplementary material for: Extracellular electrons transferred from honey probiotic Bacillus circulans inhibits inflammatory acne vulgaris
Source: Sci Rep. 2022 Nov 10;12:19217. doi: 10.1038/s41598-022-23848-9 (PMC9649788; doi:10.1038/s41598-022-23848-9)
Supplement: Supplementary file 1 — Supplementary Information. [file 41598_2022_23848_MOESM1_ESM.docx]

**Supplemental Information**

**Extracellular Electrons Transferred from Honey Probiotic *Bacillus circulans* Inhibits Inflammatory acne vulgaris**

Hsin-Jou Kao^1^, Arun Balasubramaniam^1^, Chun-Chuan Chen^1*^, and Chun-Ming Huang^1^

^1^ Department of Biomedical Sciences and Engineering, National Central University, Zhongli District, Taoyuan City, Taiwan (R.O.C.)

***** Correspondence: cchen@ncu.edu.tw; Tel.: +886-3-422-7151#27757; National Central University, No. 300, Zhongda Rd., Zhongli District, Taoyuan 32001, Taiwan (R.O.C.)

**Table S1.** 16S rRNA gene sequence of the isolated three bacteria shares 99% similarity in GenBank (https://www.ncbi.nlm.nih.gov/genbank/).

| Bacteria  name | 16S rRNA  sequence | %  identify |
| --- | --- | --- |
| *Bacillus circulans* | CTCAGGACGAACGCTGGCGGCGTGCCTAATACATGCAAGTCGAGCGGACTTTAAAAGCTTGCTTTTAAAGTTAGCGGCGGACGGGTGAGTAACACGTGGGCAACCTGCCTGTAAGACTGGGATAACTTCGGGAAACCGGAGCTAATACCGGATAATTCTTTTCATCACATGATGGAAAGCTGAAAGACGGCATCTCGCTGTCACTTACAGATGGGCCCGCGGCGCATTAGCTAGTTGGTGAGGTAACGGCTCACCAAGGCAACGATGCGTAGCCGACCTGAGAGGGTGATCGGCCACACTGGGACTGAGACACGGCCCAGACTCCTACGGGAGGCAGCAGTAGGGAATCTTCCGCAATGGACGAAAGTCTGACGGAGCAACGCCGCGTGAGTGATGAAGGTTTTCGGATCGTAAAGCTCTGTTGTTAGGGAAGAACAAGTACAAGAGTAACTGCTTGTACCTTGACGGTACCTAACCAGAAAGCCACGGCTAACTACGTGCCAGCAGCCGCGGTAATACGTAGGTGGCAAGCGTTGTCCGGAATTATTGGGCGTAAAGCGCGCGCAGGCGGTCCTTTAAGTCTGATGTGAAAGCCCACGGCTCAACCGTGGAGGGTCATTGGAAACTGGGGGACTTGAGTGCAGAAGAGAAGAGTGGAATTCCACGTGTAGCGGTGAAATGCGTAGAGATGTGGAGGAACACCAGTGGCGAAGGCGACTCTTTGGTCTGTAACTGACGCTGAGGCGCGAAAGCGTGGGGAGCAAACAGGATTAGATACCCTGGTAGTCCACGCCGTAAACGATGAGTGCTAAGTGTTAGAGGGTTTCCGCCCTTTAGTGCTGCAGCAAACGCATTAAGCACTCCGCCTGGGGAGTACGGCCGCAAGGCTGAAACTCAAAGGAATTGACGGGGGCCCGCACAAGCGGTGGAGCATGTGGTTTAATTCGAAGCAACGCGAAGAACCTTACCAGGTCTTGACATCCTCTGACACTCCTAGAGATAGGACGTTCCCCTTCGGGGGACAGAGTGACAGGTGGTGCATGGTTGTCGTCAGCTCGTGTCGTGAGATGTTGGGTTAAGTCCCGCAACGAGCGCAACCCTTGATCTTAGTTGCCAGCATTAAGTTGGGCACTCTAAGGTGACTGCCGGTGACAAACCGGAGGAAGGTGGGGATGACGTCAAATCATCATGCCCCTTATGACCTGGGCTACACACGTGCTACAATGGATGGTACAAAGGGCAGCAAAACCGCGAGGTCGAGCAAATCCCATAAAACCATTCTCAGTTCGGATTGTAGGCTGCAACTCGCCTACATGAAGCTGGAATCGCTAGTAATCGCGGATCAGCATGCCGCGGTGAATACGTTCCCGGGCCTTGTACACACCGCCCGTCACACCACGAGAGTTTGTAACACCCGAAGTCGGTGGGGTAACCTTT | 99.1 |
| *Lysinibacillus*  *fusiformis* | TCAGGACGAACGCTGGCGGCGTGCCTAATACATGCAAGTCGAGCGAACAGAGAAGGAGCTTGCTCCTTCGACGTTAGCGGCGGACGGGTGAGTAACACGTGGGCAACCTACCTTATAGTTTGGGATAACTCCGGGAAACCGGGGCTAATACCGAATAATCTGTTTCACCTCATGGTGAAACACTGAAAGACGGTTTCGGCTGTCGCTATAGGATGGGCCCGCGGCGCATTAGCTAGTTGGTGAGGTAACGGCTCACCAAGGCGACGATGCGTAGCCGACCTGAGAGGGTGATCGGCCACACTGGGACTGAGACACGGCCCAGACTCCTACGGGAGGCAGCAGTAGGGAATCTTCCACAATGGGCGAAAGCCTGATGGAGCAACGCCGCGTGAGTGAAGAAGGATTTCGGTTCGTAAAACTCTGTTGTAAGGGAAGAACAAGTACAGTAGTAACTGGCTGTACCTTGACGGTACCTTATTAGAAAGCCACGGCTAACTACGTGCCAGCAGCCGCGGTAATACGTAGGTGGCAAGCGTTGTCCGGAATTATTGGGCGTAAAGCGCGCGCAGGTGGTTTCTTAAGTCTGATGTGAAAGCCCACGGCTCAACCGTGGAGGGTCATTGGAAACTGGGAGACTTGAGTGCAGAAGAGGATAGTGGAATTCCAAGTGTAGCGGTGAAATGCGTAGAGATTTGGAGGAACACCAGTGGCGAAGGCGACTATCTGGTCTGTAACTGACACTGAGGCGCGAAAGCGTGGGGAGCAAACAGGATTAGATACCCTGGTAGTCCACGCCGTAAACGATGAGTGCTAAGTGTTAGGGGGTTTCCGCCCCTTAGTGCTGCAGCTAACGCATTAAGCACTCCGCCTGGGGAGTACGGTCGCAAGACTGAAACTCAAAGGAATTGACGGGGGCCCGCACAAGCGGTGGAGCATGTGGTTTAATTCGAAGCAACGCGAAGAACCTTACCAGGTCTTGACATCCCGTTGACCACTGTAGAGATATGGTTTCCCCTTCGGGGGCAACGGTGACAGGTGGTGCATGGTTGTCGTCAGCTCGTGTCGTGAGATGTTGGGTTAAGTCCCGCAACGAGCGCAACCCTTGATCTTAGTTGCCATCATTTAGTTGGGCACTCTAAGGTGACTGCCGGTGACAAACCGGAGGAAGGTGGGGATGACGTCAAATCATCATGCCCCTTATGACCTGGGCTACACACGTGCTACAATGGACGATACAAACGGTTGCCAACTCGCGAGAGGGAGCTAATCCGATAAAGTCGTTCTCAGTTCGGATTGTAGGCTGCAACTCGCCTACATGAAGCCGGAATCGCTAGTAATCGCGGATCAGCATGCCGCGGTGAATACGTTCCCGGGCCTTGTACACACCGCCCGTCACACCACGAGAGTTTGTAACACCCGAAGTCGGTGAGGTAACCTTTTGGAGCCAGCCGCC | 99.9 |
| *Bacillus asahii* | TCAGGACGAACGCTGGCGGCGTGCCTAATACATGCAAGTCGAGCGAATCAATGGGAGCTTGCTCCCTGAGATTAGCGGCGGACGGGTGAGTAACACGTGGGTAACCTGCCTGTAAGACTGGGATAACTTCGGGAAACCGGAGCTAATACCGGATAGTTTCTTTTCTCGCATGAGAGAAGATGGAAAGATGGTTTCGGCTATCACTTACAGATGGACCCGCGGCGCATTAGCTAGTTGGTGAGGTAACGGCTCACCAAGGCGACGATGCGTAGCCGACCTGAGAGGGTGATCGGCCACACTGGGACTGAGACACGGCCCAGACTCCTACGGGAGGCAGCAGTAGGGAATCTTCCGCAATGGACGAAAGTCTGACGGAGCAACGCCGCGTGAGCGAAGAAGGCCTTCGGGTCGTAAAGCTCTGTTGTTAGGGAAGAACAAGTACGAGAGTAACTGCTCGTACCTTGACGGTACCTAACCAGAAAGCCACGGCTAACTACGTGCCAGCAGCCGCGGTAATACGTAGGTGGCAAGCGTTGTCCGGAATTATTGGGCGTAAAGCGCGCGCAGGCGGTCCTTTAAGTCTGATGTGAAAGCCCACGGCTCAACCGTGGAGGGTCATTGGAAACTGGGGGACTTGAGTGCAGAAGAGGAGAGTGGAATTCCACGTGTAGCGGTGAAATGCGTAGAGATGTGGAGGAACACCAGTGGCGAAGGCGACTCTCTGGTCTGTAACTGACGCTGAGGCGCGAAAGCGTGGGGAGCAAACAGGATTAGATACCCTGGTAGTCCACGCCGTAAACGATGAGTGCTAAGTGTTAGAGGGTTTCCGCCCTTTAGTGCTGCAGCTAACGCATTAAGCACTCCGCCTGGGGAGTACGGCCGCAAGGCTGAAACTCAAAGGAATTGACGGGGGCCCGCACAAGCGGTGGAGCATGTGGTTTAATTCGAAGCAACGCGAAGAACCTTACCAGGTCTTGACATCCTCTGCCAACCCTAGAGATAGGGCGTTCCCCTTCGGGGGACAGAGTGACAGGTGGTGCATGGTTGTCGTCAGCTCGTGTCGTGAGATGTTGGGTTAAGTCCCGCAACGAGCGCAACCCTTGATCTTAGTTGCCAGCATTCAGTTGGGCACTCTAAGGTGACTGCCGGTGACAAACCGGAGGAAGGTGGGGATGACGTCAAATCATCATGCCCCTTATGACCTGGGCTACACACGTGCTACAATGGATGGTACAAAGAGCTGCGAACCCGCGAGGGTAAGCGAATCTCATAAAGCCATTCTCAGTTCGGATTGTAGGCTGCAACTCGCCTACATGAAGCCGGAATCGCTAGTAATCGCGGATCAGCATGCCGCGGTGAATACGTTCCCGGGCCTTGTACACACCGCCCGTCACACCACGAGAGTTTGTAACACCCGAAGTCGGTGAGGTAACCTTTTGGAGCCAGCCGCCTA | 99.6 |

**
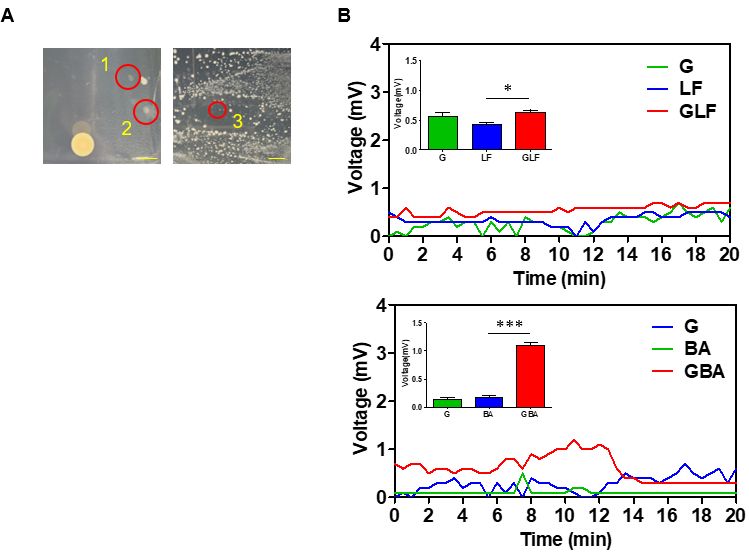
**

**Figure S1.** A) Bacteria was isolated from TSB agar plate with 10% honey. (1) *Bacillus circulans*, (2) *Lysinibacillus fusiformis*, and (3) *Bacillus asahii.* B) Voltage changes detection (mV) for 20 min in media containing glucose (G), *L. fusiformis* (LF), *B. asahii* (BA), *L.* *fusiformis* plus glucose (GLF) or *B. asahii* plus glucose (GBA). Data are expressed as the mean ± SD of three separate experiments. **p* < 0.05 and ****p* < 0.001. Scar bar 7.5mm.


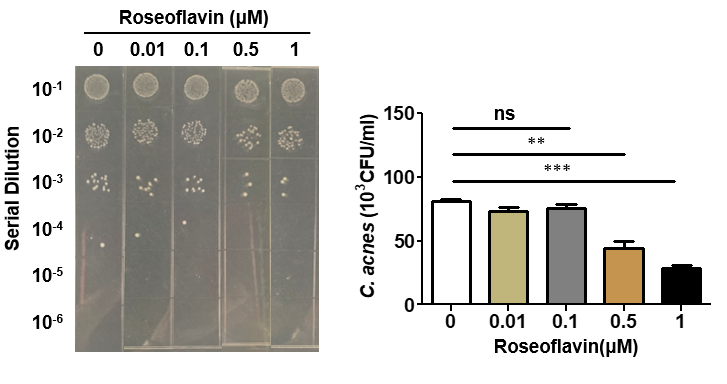


**Figure S2.** The efficacy of different concentrations roseoflavin influences *C. acnes* growth. Data are expressed as the mean ± SD of three separate experiments. ***p* < 0.01 and ****p* < 0.001 and ns = non-significant.
